# Knockdown of the partner protein OsNAR2.1 for high-affinity nitrate transport represses lateral root formation in a nitrate-dependent manner

**DOI:** 10.1038/srep18192

**Published:** 2015-12-08

**Authors:** Shuangjie Huang, Si Chen, Zhihao Liang, Chenming Zhang, Ming Yan, Jingguang Chen, Guohua Xu, Xiaorong Fan, Yali Zhang

**Affiliations:** 1 State Key Laboratory of Crop Genetics and Germplasm Enhancement, Key Laboratory of Plant Nutrition and Fertilization in Low-Middle Reaches of the Yangtze River, Ministry of Agriculture, Nanjing Agricultural University, Nanjing 210095, P.R. China

## Abstract

The morphological plasticity of root systems is critical for plant survival, and understanding the mechanisms underlying root adaptation to nitrogen (N) fluctuation is critical for sustainable agriculture; however, the molecular mechanism of N-dependent root growth in rice remains unclear. This study aimed to identify the role of the complementary high-affinity NO_3_^−^ transport protein OsNAR2.1 in NO_3_^−^-regulated rice root growth. Comparisons with wild-type (WT) plants showed that knockdown of OsNAR2.1 inhibited lateral root (LR) formation under low NO_3_^−^ concentrations, but not under low NH_4_^+^ concentrations. ^15^N-labelling NO_3_^−^ supplies (provided at concentrations of 0–10 mM) demonstrated that (i) defects in LR formation in mutants subjected to low external NO_3_^−^ concentrations resulted from impaired NO_3_^−^ uptake, and (ii) the mutants had significantly fewer LRs than the WT plants when root N contents were similar between genotypes. LR formation in *osnar2*.*1* mutants was less sensitive to localised NO_3_^−^ supply than LR formation in WT plants, suggesting that OsNAR2.1 may be involved in a NO_3_^−^-signalling pathway that controls LR formation. Knockdown of OsNAR2.1 inhibited LR formation by decreasing auxin transport from shoots to roots. Thus, OsNAR2.1 probably functions in both NO_3_^−^ uptake and NO_3_^−^-signalling.

Plants have diverse mechanisms for adapting to nutrient supply conditions. Among these mechanisms, the plasticity of root development is vital. Lateral roots (LRs) are generally more sensitive to variations in nutrient conditions compared to primary roots^1^,^2^. Lateral root development begins with the initiation of founder cells in the root pericycle just behind the primary root apex and continues with the formation of a cluster of cells constituting the LR primordium, followed by the formation of a radially symmetrical meristem^3^.

Nitrogen (N) is an essential macronutrient for plant growth and crop productivity. Changes in N availability in the nutrient medium induce plasticity in LR initiation and elongation^4^,^5^,^6^,^7^,^8^,^9^,^10^. Lateral root development in response to NO_3_^−^ has been investigated extensively in the dicot model plant *Arabidopsis*^5^,^10^,^11^,^12^,^13^. A striking example of plasticity in LR development is seen in *Arabidopsis* responding to localised NO_3_^−^ treatment through stimulation of LR elongation^5^. Studies of an *Arabidopsis* nitrate reductase double mutant suggested that the local stimulation of LR elongation is a consequence of the NO_3_^−^ ion acting as a signal rather than as a nutrient. *AtANR1* (a NO_3_^−^-inducible gene) and *AtNRT1*.*1* (*CHL1*/*NPF6*.*3*) genes, which encode a transcription factor and a dual NO_3_^−^ transporter, respectively, were proposed to consecutively control the stimulatory effect of NO_3_^−^ on LR elongation^5^,^6^,^11^. The positive regulatory role of *ANR1* in LR elongation was recently corroborated when overexpression in transgenic lines stimulated LR elongation whilst having no direct effect on LR density or primary root growth^14^. In addition to local NO_3_^−^ responses, LR growth is stimulated in response to mild N deficiency and suppressed under excess N supply by systemic plant signals carrying information on the nutritional status of distant plant organs^1^,^6^,^15^,^16^,^17^,^18^. Recent studies have highlighted the role of *AtNRT2*.*1* in root architecture response to low NO_3_^−^ availability, especially in LR initiation^16^,^19^. Experiments using *atnar2*.*1-1* identified the role of AtNAR2.1 in LR responses to low NO_3_^−^ supply^20^; further work showed that the essential role of AtNAR2.1 for the localization of AtNRT2.1 to the plasma membrane^20^,^21^. miR393/AFB3 is a NO_3_^−^-responsive module that controls LR density in responses to external and internal N concentrations in *Arabidopsis*^12^,^13^. Interestingly, AGL7-clade MADS-box gene *AtAGL21* plays a crucial role in both LR formation and elongation^22^. Thus, NO_3_^−^-dependent root development is apparently under the control of complex mechanisms, although its signalling components have remained largely unidentified.

Our understanding of NO_3_^−^-regulated LR development is limited in monocot plants, including rice, the model cereal plant (*Oryza sativa* L.)^8^,^23^. Elevated NO_3_^−^ responsiveness in rice plants is strongly related to increased LR initiation regardless of whether the roots system was split or whole^8^,^9^. Recent work showed that the transcriptional levels of four *ANR1*-like genes (*OsMADS25*, *OsMADS27*, *OsMADS57* and *OsMADS61*) were markedly modulated by NO_3_^−^ availability^24^. Furthermore, in miR444a-overexpressing rice lines expression of the target *ANR1*-like genes was down-regulated and LR elongation was less responsive to localised NO_3_^−^ 23. These studies indicated that *ANR1*-like genes might have a similar role in regulating root developmental response to NO_3_^−^ in rice, despite the evolutionary distance between *Arabidopsis* and rice.

We previously reported that *OsNAR2*.*1*, a rice *NAR2*-like gene, has no known transport activity, but is required to complement high-affinity NO_3_^−^ transport. Yeast two-hybridisation showed that OsNAR2.1 interacted with OsNRT2.1/2.2/2.3a, and knockdown of OsNAR2.1 suppressed expression of the three high-affinity-transport- system NO_3_^−^ transporters, unlike OsNRT1.1^25^,^26^,^27^,^28^. Knockdown of OsNAR2.1 impaired NO_3_^−^ uptake; mutants had only ~65% of the N concentration measured in wild-type (WT) plants when the NO_3_^−^ supply was limited. No such difference was found when a low concentration of NH_4_^+^ was provided as the sole inorganic N source^27^. In this study, we confirmed inhibition of LR formation in the *osnar2*.*1* mutants (compared to WT plants) under low NO_3_^−^ concentrations. ^15^N-labelling of NO_3_^−^ supplies (provided at concentrations of 0–10 mM) and our localised-NO_3_^−^ treatments showed that defective LR formation in *OsNAR2*.*1* knockdown plants may be due (i) an impairment of NO_3_^−^ uptake, and (ii) a NO_3_^−^-signalling function.

## Results

### Knockdown of OsNAR2.1 inhibited root growth when N was supplied as NO_3_^−^

**Figure 1:**
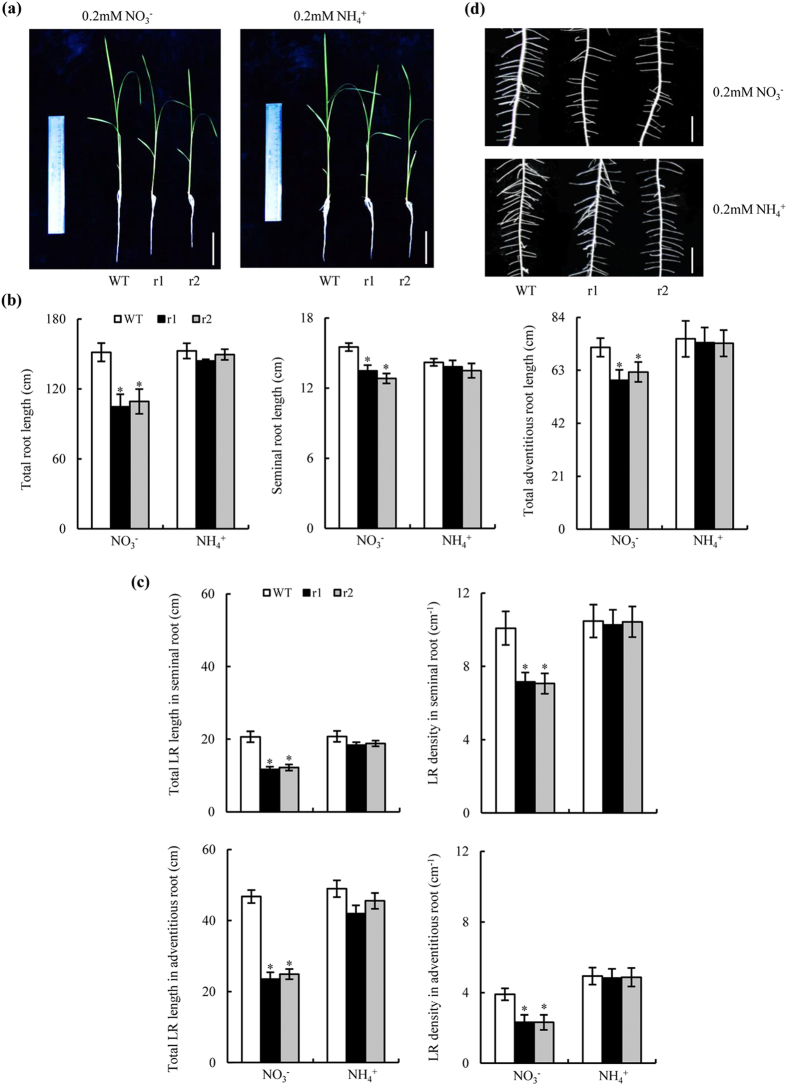
Root morphology of wild-type (WT) and *osnar2*.*1* knockdown lines (r1 and r2). Rice seedlings were grown for 1 wk in hydroponic media containing 0.2 mM NO_3_^−^ or NH_4_^+^. (**a**) Morphology of rice plants (bar = 5 cm); (**b**) Total root length (including the seminal root, adventitious roots and lateral roots [LR]); (**c**) LR morphology in seminal and adventitious roots (bar = 1 cm). (**d**) Morphology of LRs on the seminal root. Values are means ± SE (n = 6). *P < 0.05 (ANOVA) comparing WT plants and two mutant lines in the same treatment.

Total root lengths were shorter in *osnar2*.*1* mutants than in WT plants at a NH_4_^+^:NO_3_^−^ concentration ratio of 25:75. The difference in root length between the genotypes was greatest when the ionic ratio was 0:100 (Supplementary Fig. S1 online); mutant line total root lengths were *ca*. 30% of those in WT plants at this concentration ratio. However, total root lengths were similar between genotypes when NH_4_^+^-N was supplied (Fig. 1a,b). Reductions in total root lengths in the mutants were largely attributable to reduced total LR lengths (Fig. 1c). The total LR lengths of seminal and adventitious roots in the RNAi lines were *ca*. 64 and 53%, respectively, of those in WT plants when NO_3_^−^-N was supplied. The LR density in the RNAi lines was reduced to a greater extent than the mean LR length (Fig. 1c, Supplementary Fig. S2). The LR densities of the seminal and adventitious roots in the two *osnar2*.*1* mutants were reduced by *ca*. 29% and 40%, respectively, in comparison with those in the WT plants (Fig. 1d). The lengths of the seminal and all adventitious roots in the *osnar2*.*1* mutants were *ca*. 15% shorter than those of WT plants when NO_3_^−^-N was supplied (Fig. 1b). However, the numbers and mean lengths of adventitious roots were not different from those of WT plants (Supplementary Fig. S3 online).

### Knockdown of OsNAR2.1 repressed LR initiation when N was supplied as NO_3_^−^

**Figure 2:**
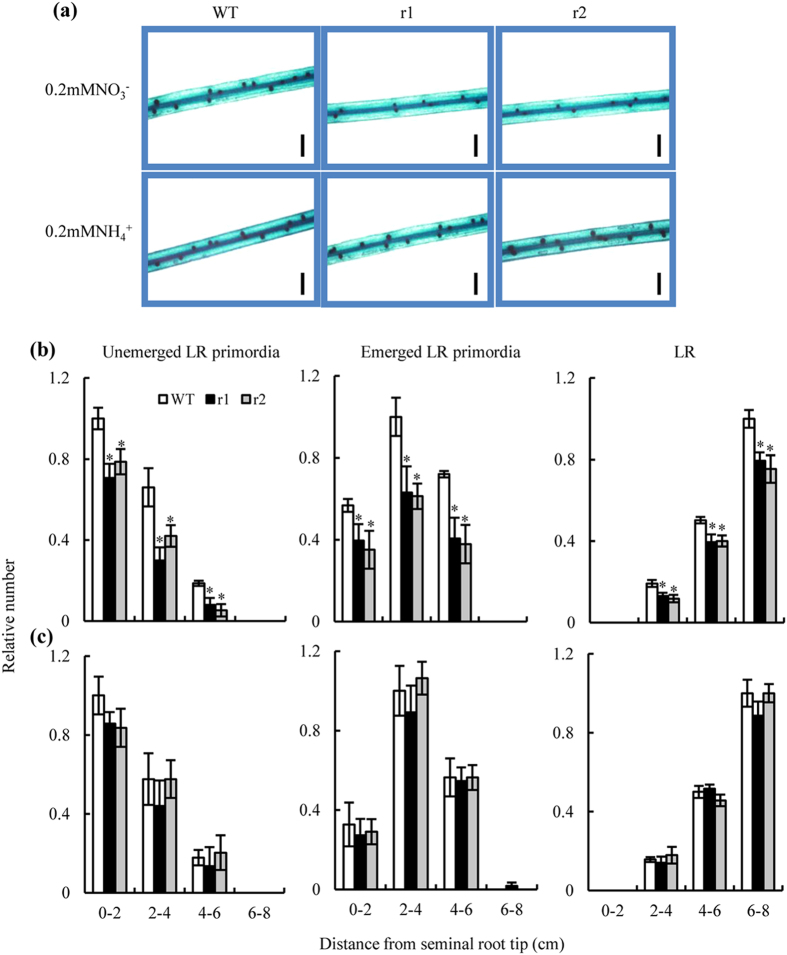
Lateral root (LR) development on the seminal roots of wild-type (WT) and *osnar2*.*1* knockdown lines (r1 and r2). LR development in the seminal roots was measured after the seedlings had been grown for 1 wk in hydroponic media containing 0.2 mM NO_3_^−^ or NH_4_^+^. (**a**) LR primordia in 2–4 cm seminal root from the root tip (bar = 1 mm); (**b,c**) Microscopic images of LR development at a concentration of 0.2 mM NO_3_^−^ (**b**) or 0.2 mM NH_4_^+^ (**c**). Relative numbers for three LR stages were normalised to the largest number in WT plants. Values are means ± SE (n = 6). *P < 0.05 (ANOVA) comparing WT plants and two mutant lines in the same root zone.

Two-week-old rice seedlings had seminal roots that were longer than the adventitious roots. Our preliminary experiment showed that the response of seminal root LRs to N treatment was similar to that of adventitious root LRs. We therefore chose seminal roots to represent root responses in our study of the effects of OsNAR2.1 on the rice root system. Compared to WT plants, the number of LR primordia was reduced markedly in the RNAi lines when NO_3_^−^-N was supplied (Fig. 2a). When NH_4_^+^-N was supplied, we observed no difference in LR development between the WT and the mutants (Fig. 2a,c). When NO_3_^−^- N was supplied, our microscopic observations detected significant differences between genotypes in (i) the numbers of unemerged and emerged LR primordia and (ii) the numbers of LRs at distances 0–8 cm behind the seminal root tips (Fig. 2b). A marked decrease in the number of LR primordia (number of primordia from the first division of the pericycle cells to emergence) resulted in a 23% reduction in the LR numbers in 6–8 cm-lengths of roots in the mutants (compared to roots in the WT plants) after incubation for 7 d with a NO_3_^−^-N supply.

### N accumulation in the *osnar2*.*1* mutants was inhibited across a wide range of NO_3_^−^ concentrations

**Figure 3:**
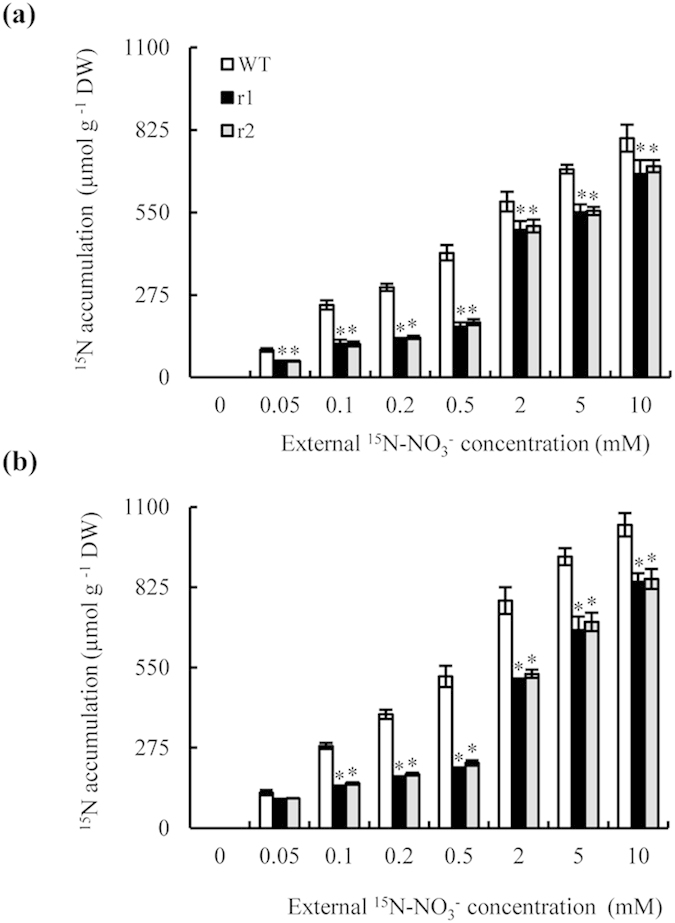
Cumulative ^15^N-NO_3_^−^ uptake in wild-type (WT) and *osnar2*.*1* knockdown lines (r1 and r2). Rice seedlings were grown for 1 wk in hydroponic medium containing 10 mM NO_3_^−^ and then transferred for a further week to media containing 0–10 mM ^15^N-NO_3_^−^. (a, b) Cumulative ^15^N-NO_3_^−^ uptake in the shoots (**a**) and roots (**b**). Values are means ± SE (n = 6). *P < 0.05 (ANOVA) comparing WT plants and two mutant lines in the same treatment.

We determined whether defective LR formation in the *osnar2*.*1* mutants under NO_3_^−^ supply was attributable to the inhibition of N uptake, by quantifying the level of N limitation experienced by rice seedlings through measurements of the cumulative uptake of ^15^N-NO_3_^−^ during the entire 7 d period following transfer to media containing a range of labelled NO_3_^−^ concentrations (Fig. 3a,b). Compared to WT plants, the mutants had reduced N accumulations in the shoots and the roots; reductions ranged between 61% and 18% at external NO_3_^−^ concentrations of 0.05–10 mM.

### Lateral root formation responses to reduced root N accumulation

**Figure 4:**
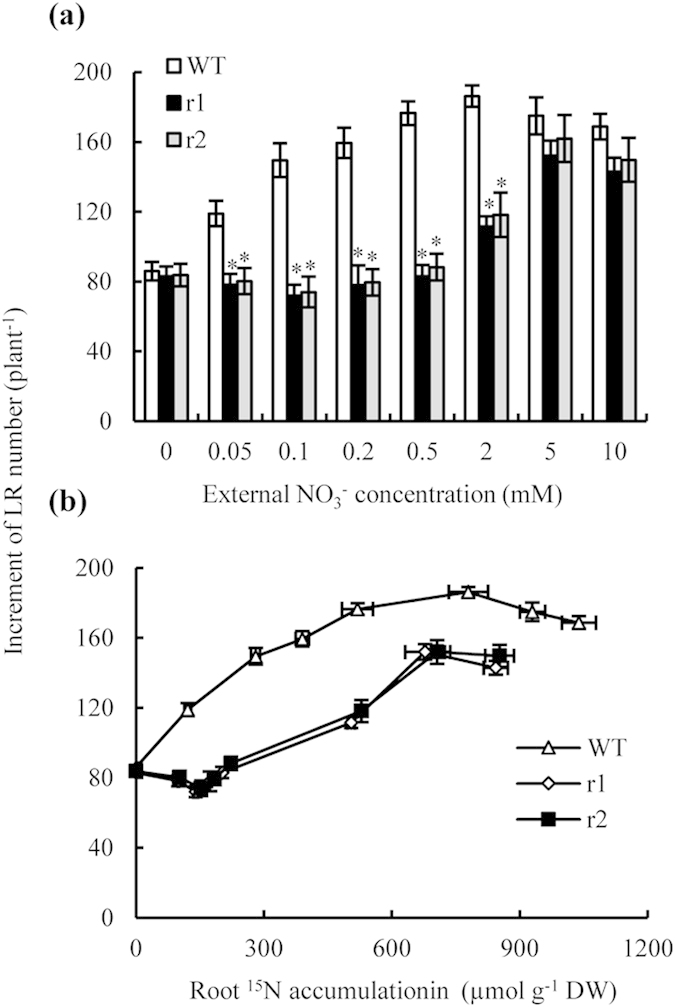
Changes in lateral root (LR) numbers in response to varying external and internal nitrate concentrations in wild-type (WT) and *osnar2*.*1* knockdown lines (r1 and r2). Rice seedlings were grown for 1 wk in hydroponic medium containing 10 mM NO_3_^−^ and then transferred for a further week to media containing 0-10 mM ^15^N-NO_3_^−^. (**a**) LR number responding to varying external NO_3_^−^ concentrations; (**b**) LR number responding to varying internal ^15^N accumulation levels in the roots. Values are means ± SE (n = 6). *P < 0.05 (ANOVA) comparing WT plants and two mutant lines in the same treatment.

We investigated LR formation in rice plants after transfer from medium containing 10 mM NO_3_^−^ to media containing a range of NO_3_^−^ concentrations (0–10 mM). After 7 d, the elongation of seedling seminal and adventitious roots had responded little to the treatments (Fig. 1, Supplementary Fig. S3), but subsequent LR formation was strongly affected by external NO_3_^−^ concentrations (Fig. 4a). Lateral root numbers on the seminal roots of WT plants increased with increasing external NO_3_^−^ concentration (0.05–2 mM), but decreased with continued increases in external NO_3_^−^ concentrations above 2 mM (Fig. 4a), in agreement with our previous findings^29^,^30^.

Lateral root development is modulated by internal plant N status^11^,^16^,^18^,^31^. A plot of the number of new LRs formed during the experimental period against the internal root N content in WT plants (Fig. 4b) showed clearly that LR numbers increased with internal root N content. N accumulation increased when the concentration in the root was less than *ca*. 780 μmol g^−1^ (external NO_3_^−^ ≤ 2 mM); however, LR formation was inhibited when the internal N content in the root was greater than *ca*. 780 μmol g^−1^ (external NO_3_^−^ ≥ 2 mM). A similar trend was detected when we plotted the number of newly formed LRs in the *osnar2*.*1* knockdown mutants against a range of internal root N contents. These results suggest that the inhibition of LR initiation in *osnar2*.*1* mutants at a low NO_3_^−^ concentrations is due to impaired NO_3_^−^ uptake. However, defects in LR formation of *osnar2*.*1* mutants were not completely explainable by reduced NO_3_^−^ uptake because when internal N concentrations in the roots of WT plants and mutants were similar, the mutants still had fewer visible LRs than the WT plants.

### LR formation was less sensitive to localised NO_3_^−^ supply in the *osnar2*.*1* mutants than in WT plants

**Figure 5:**
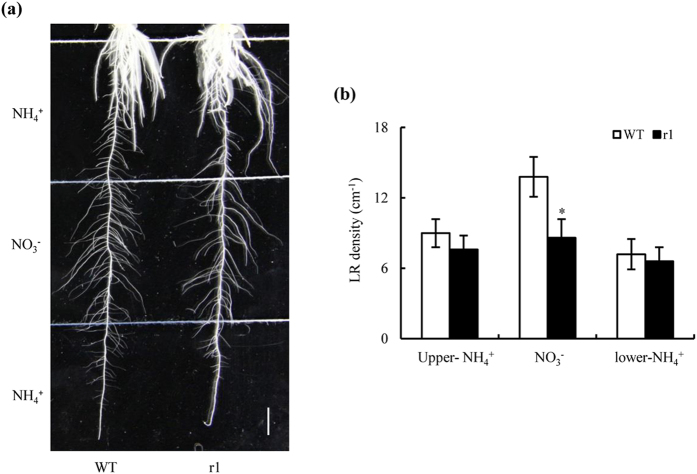
Effect of a localised NO_3_^−^ supply on lateral root (LR) development in wild-type (WT) and *osnar2*.*1* knockdown line (r1). Rice seeds were germinated in trays over a period of 2 d and then transferred to plant culture dishes containing 0.6% Phytagel™ media. Culture dishes were divided into three 5-cm segments; the upper and lower segments were supplemented with 0.2 mM NH_4_^+^, and the middle segment was supplemented with 0.2 mM NO_3_^−^. (**a**) Image of LRs responding to localised NO_3_^−^ supply after a 3 wk growth period. Bar = 1 cm; (**b**) LR density in three segments. Values are means ± SE (n = 8). *P < 0.05 (ANOVA) comparing WT and r1 plants in the same treatment.

Our data to this point suggested that OsNAR2.1 may be involved in a signalling function; we therefore examined the responses of seminal root LRs to localised NO_3_^−^ supply in WT plants and *osnar2*.*1* mutants. LR densities on seminal roots provided with a localised NO_3_^−^ supply were 33% lower in *osnar2*.*1* mutants than in WT plants (Fig. 5a,b). However, LR densities were similar on seminal roots of mutants and WT plants when we provided a localised NH_4_^+^ supply. These data suggest that a knockdown of OsNAR2.1 inhibited LR formation via a NO_3_^−^-signalling pathway when NO_3_^−^-N was supplied.

### Knockdown of OsNAR2.1 inhibited auxin transport from the shoot to the root when N was supplied as NO_3_^−^

**Figure 6:**
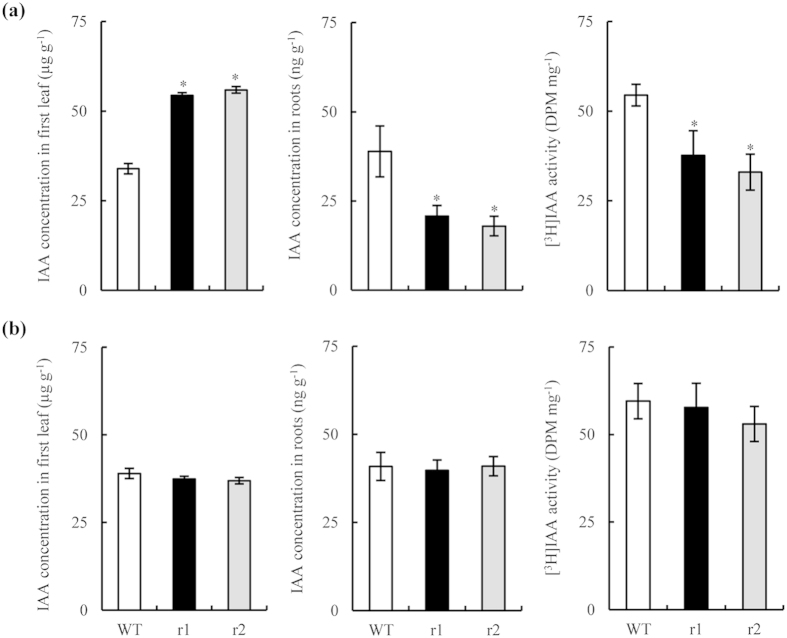
IAA concentration in the first leaf and roots and [^3^H]IAA transport in wild-type (WT) and *osnar2*.*1* knockdown lines (r1 and r2). Seedlings were grown hydroponically for 1 wk in nutrient solution containing 0.2 mM NO_3_^−^ (**a**) or NH_4_^+^ (**b**). Values are means ± SE (n = 6). *P < 0.05 (ANOVA) comparing WT plants and two mutant lines.

Several plant hormones control LR formation; auxin plays a pivotal role^32^,^33^. To determine whether auxin affected LR formation in *osnar2*.*1* mutants, we analysed endogenous IAA concentrations in the first leaf and in the root. IAA concentrations in the first leaf of the two *osnar2*.*1* mutants supplied with NO_3_^−^ were elevated by *ca*. 62% in comparison with WT seedlings; conversely, IAA concentrations in the roots of two *osnar2*.*1* mutants were *ca*. 50% lower than those in WT plants (Fig. 6a). When NH_4_^+^-N was supplied, auxin distributions were similar between genotypes (Fig. 6b). Thus, knockdown of OsNAR2.1 probably inhibited auxin polar transport from shoots to roots when NO_3_^−^-N was supplied. We therefore conducted [^3^H]IAA transport assays. When NO_3_^−^-N was supplied to the two mutant seedlings, [^3^H]IAA transport from their shoots to their roots was reduced significantly, and the [^3^H]IAA activity in the roots was reduced in consequence. These data confirm that auxin polar transport from shoots to roots was inhibited in *osnar2*.*1* mutants supplied with NO_3_^−^ as an N source.

### Exogenous application of NAA recovered LR initiation in *osnar2*.*1* mutants

**Figure 7:**
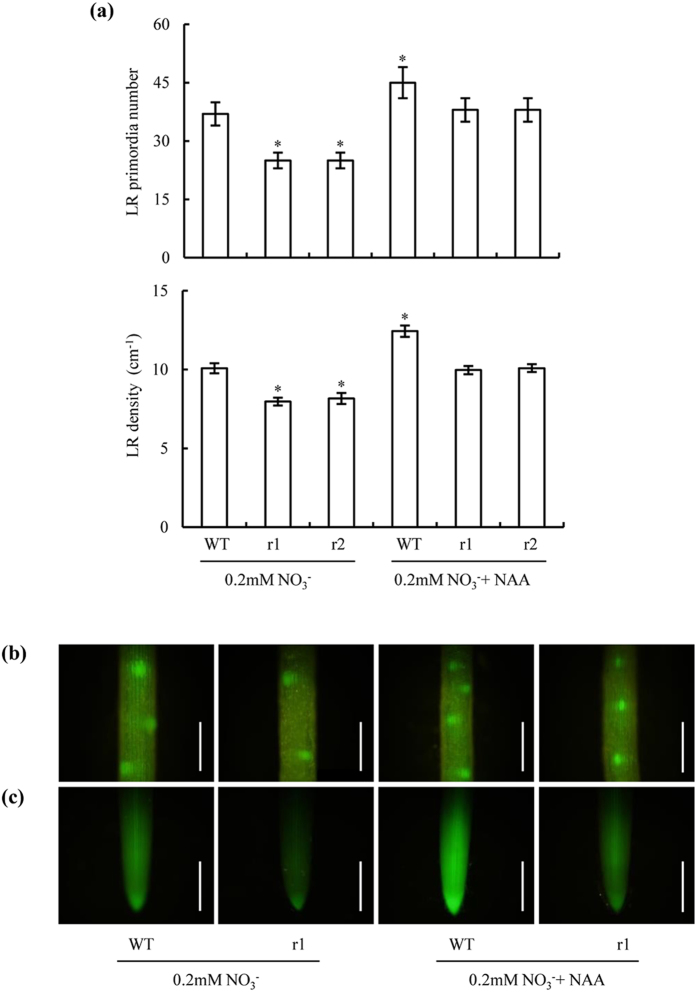
Lateral root (LR) primordium numbers and LR density in seminal roots of wild-type (WT) and *osnar2*.*1* knockdown lines (r1 and r2). Seedlings were grown for 1 wk in nutrient solution containing 0.2 mM NO_3_^−^ with or without NAA application (1 nM) in agar media. (**a**) LR primordium numbers and LR density in seminal roots; (**b**) *DR5::GFP* expression in the LR primordium; (**c**) *DR5::GFP* expression in the seminal root tips. Bar = 1 mm. Values are means ± SE (n = 6). *P < 0.05 (ANOVA) comparing WT plants subjected to 0.2 mM NO_3_^−^ and five other treatments.

Application of NAA (1 nM) counteracted the effects of OsNAR2.1 knockdown on LR primordium numbers and LR density when N was supplied as NO_3_^−^ (Fig. 7a,b). LR initiation in *osnar2*.*1* mutants supplied with NO_3_^−^ and treated with NAA was similar to LR initiation in WT plants supplied with NO_3_^−^ as the N source. Furthermore, exogenous NAA application enhanced LR initiation in WT rice seedlings supplied with NO_3_^−^. These findings were concordant with changes in *DR5::GFP* expression levels in seminal root tips (Fig. 7c). Thus, reduced IAA concentrations in the two *osnar2*.*1* mutants played a key role in the inhibition of LR initiation.

### Knockdown of OsNAR2.1 reduced expression levels of *PINs* in the roots

**Figure 8:**
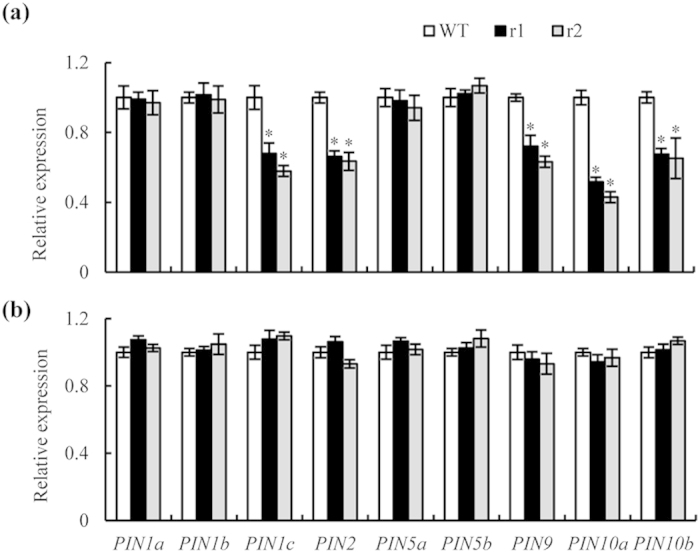
qRT-PCR analysis of *PIN* family genes in wild-type (WT) and *osnar2*.*1* knockdown lines (r1 and r2). Seedlings were grown hydroponically for 1 wk in nutrient solution containing 0.2 mM NO_3_^−^ or NH_4_^+^. (**a,b**) Expression levels in medium containing (**a**) 0.2 mM NO_3_^−^ or (**b**) 0.2 mM NH_4_^+^. Relative mRNA levels for individual genes were normalised relative to *OsACT*. Values are means ± SE (n = 4). *P < 0.05 (ANOVA) comparing expression levels of the same gene in WT plants and two mutant lines.

Most auxin transport occurs via the polar transport stream, which is facilitated by proteins of the PIN family^34^. Our qRT-PCR analysis showed that the expression levels of *PIN1c*, *PIN2*, *PIN9*, and *PIN10a-b* in the roots subjected to NO_3_^−^ treatments were significantly lower in the two *osnar2*.*1* mutants than in the WT rice seedlings (Fig. 8a). When NH_4_^+^-N was supplied, *PIN* expression levels were similar between the two *osnar2*.*1* mutants and the WT (Fig. 8b).

## Discussion

Mounting evidence shows that LR development is highly plastic and responsive to N treatments^1^,^4^,^11^,^17^,^20^,^29^,^30^,^35^. The striking effects of localised-NO_3_^−^ and high NO_3_^−^ media on LR growth have been investigated extensively in the model plant *Arabidopsis*^5^,^10^,^11^,^15^; however, the mechanism of N-dependent LR growth in rice remains unclear.

We observed inhibition of LR formation in the *osnar2*.*1* mutants (compared to WT plants) subjected to low NO_3_^−^ concentrations, but not in those subjected to low NH_4_^+^ concentrations. Knockdown of OsNAR2.1 reportedly impairs NO_3_^−^ uptake at a NO_3_^−^ concentration of 0.2 mM; the concentration of N in the mutants was only *ca*. 65% of that in the WT plants, but no differences were observed among genotypes at an NH_4_^+^ concentration of 0.2 mM^27^. Our study is the first to determine whether the inhibition of LR initiation in *osnar2*.*1* mutants at low NO_3_^−^ concentrations is due to impaired NO_3_^−^ uptake. When we plotted LR number under varying external NO_3_^−^ concentrations against root N content, we found that trends in the relationship were similar between mutants and WT plants: LR numbers increased with root N content up to a concentration of 700–780 μmol g^−1^; above this concentration, LR numbers decreased (Fig. 4b). Under external NO_3_^−^ (^15^N-NO_3_^−^) concentrations of 5 and 10 mM, impaired NO_3_^−^ uptake in the *osnar2*.*1* mutants resulted in root N accumulations of 700–780 μmol g^−1^. Therefore, similar LR formations in WT plants and the two mutants were not unexpected at high NO_3_^−^ concentrations (Fig. 4a). Reduced root N accumulation in *osnar2*.*1* mutants subjected to low external NO_3_^−^ concentrations inhibited LR formation (in comparison with WT rice plants). Overall, these results suggest that the knockdown of *O*sNAR2.1 probably reduced LR formation under low external NO_3_^−^ concentrations via impairment of NO_3_^−^ uptake.

Interestingly, inhibition of LR growth by OsNAR2.1 knockdown cannot be fully explained by reduced N uptake in the mutants. When internal root N contents in the roots of the WT and mutant plants were similar across the entire range of N contents (i.e. under external ^15^N-NO_3_^−^ concentrations ranging from 0.05 to 10 mM), the *osnar2*.*1* mutant plants always had significantly fewer LRs than the WT plants; e.g. when internal root N contents reached 520 μmol g^−1^, LR numbers in the two mutants were 33% fewer than those in the WT plants. Furthermore, the LR formation response to localised NO_3_^−^ supply was weaker in the *osnar2*.*1* mutants than in WT plants (Fig. 5). Thus, the NO_3_^−^-responsive *OsNAR2.1* gene may be involved in a signalling pathway controlling LR formation in environments supplied with NO_3_^−^-N.

The expression levels of *OsNRT2*.*1*, *OsNRT2*.*2*, and *OsNRT2*.*3a* in the roots of two *osnar2*.*1* mutants are reportedly much reduced in comparison with the abundant expression levels in WT plants^27^. Recent evidence suggests that in the model plant *Arabidopsis*, AtNRT1.1 (CHL1) is a NO_3_^−^ sensor that activates the ANR1-mediated NO_3_^−^-signalling pathway to regulate LR proliferation^5^,^11^,^35^,^36^,^37^. Furthermore, AtNRT2.1 may have a direct stimulatory role in LR initiation under low NO_3_^−^ concentrations^19^. Interestingly, AtNAR2.1 may also participate in the signalling pathway that integrates nutritional cues for LR proliferation by targeting AtNRT2.1 at the plasma membrane^20^,^21^. Therefore, it is reasonable to postulate that the inhibition of LR initiation in the *osnar2*.*1* mutants is also related to the interaction of proteins affected by the knockdown of OsNAR2.1. The transcript levels of *OsNAR2*.*1* and the three NO_3_^−^ transporters are rapidly induced by NO_3_^−^ supply, with peak levels occurring 1–2 h after the initiation of treatments^25^,^27^,^38^,^39^. Furthermore, *OsNAR2*.*1*, *OsNRT2*.*1*, and *OsNRT2*.*2* are expressed abundantly throughout the primary and lateral roots^25^,^26^,^27^, and *OsNRT2*.*3a* is expressed abundantly in the root stelar cells, particularly in the xylem parenchyma tissue^40^. More experiments are required to elucidate the participation of OsNAR2.1 regulatory networks in rice LR formation under low NO_3_^−^ concentrations.

Plants adjust their growth and development in response to changing environmental conditions through the perception and integration of external signals into the signalling pathways of plant hormones, such as auxin^12^,^13^,^33^,^41^,^42^,^43^,^44^,^45^. Auxin plays dominant roles in the specification of the founder cells that initiate LR formation and in the later stages of LR development^32^,^33^. Diverse environmental and endogenous signals may be integrated to mediate changes in auxin distribution via effects on polar transport^46^,^47^. Lateral root growth is not stimulated by localised NO_3_^−^ supply in the auxin-insensitive mutant *axr4*, which suggests an overlap between the auxin and NO_3_^−^ signalling pathways^6^. These two signalling pathways are further linked through an effect on auxin transport via AtNRT1.1^37^.

In our study, (i) elevated IAA concentrations in mutant leaves, (ii) reduced IAA concentrations in mutant roots, and (iii) repressed mutant [^3^H]IAA transport from the shoot to root (in comparison with WT plants) suggested that auxin polar transport was inhibited by the knockdown of OsNAR2.1. These effects were correlated with a decrease in the transcript levels of five *PIN* genes in mutant roots (relative to WT plants). Exogenous NAA application restored LR initiation and *DR5::GFP* expression levels in seminal root tips of *osnar2*.*1* mutants to levels similar to those in WT plants, further demonstrating that a reduced auxin concentration contributed to repressed LR initiation in the *osnar2*.*1* mutants. *OsNAR2*.*1* expression is dominant in roots and minimal in shoots^25^,^38^. Furthermore, in comparison with normal nutrient supply conditions, IAA transport is reduced in rice when N supply is limiting^29^,^30^. Thus, the repression of [^3^H]IAA transport from the shoot to the root in the *osnar2*.*1* mutants (relative to WT plants) under low external NO_3_^−^ concentrations can probably be attributed to N starvation system signals that inhibited auxin polar transport from the shoot to the root.

In conclusion, OsNAR2.1 had a key function in coordinating LR formation at low external NO_3_^−^ concentrations. Its effects on LR formation most likely operated through a combination of roles in both NO_3_^−^ uptake and NO_3_^−^ signalling.

## Methods

### Plant material and growth conditions

The Nipponbare rice ecotype was used as the WT. The *osnar2*.*1* mutant lines (T2 generation) obtained from RNA interference were reported in previous work^27^. Plants were grown in a greenhouse under natural light at day/night temperatures of 30 °C/18 °C. Seven-day-old seedlings of uniform size and vigour were transplanted into holes in a lid placed over the tops of 7-L pots (four holes per lid and three seedlings per hole). Nutrient solutions varying from one-quarter to half strength were applied for 2 d, and then full-strength nutrient solution was applied for a further week. Pots receiving NH_4_^+^ or NO_3_^−^ were filled with 0.2 mM N solutions. The treatment protocol for providing NO_3_^−^ concentrations of 0–10 mM to the plants followed a previously reported methodology^16^. Seven-day-old WT and mutant seedlings were transplanted into nutrient solutions varying from one-quarter to half strength for 2 d and then into nutrient solution containing 10 mM NO_3_^−^ for 1 additional wk. Rice plants were transferred to nutrient solutions containing various NO_3_^−^ (^15^N-NO_3_^−^, atom% ^15^N: 99%) concentrations (0–10 mM) for 1 wk before harvest.

The full chemical composition of the International Rice Research Institute (IRRI) nutrient solution was (mM): 0.3 KH_2_PO_4_, 0.35 K_2_SO_4_, 1.0 CaCl_2_, 1.0 MgSO_4_·7H_2_O, 0.5 Na_2_SiO_3_, and (μM) 20.0 Fe-EDTA, 9.0 MnCl_2_, 0.39 (NH_4_)_6_Mo_7_O_24_, 20.0 H_3_BO_3_, 0.77 ZnSO_4_ and 0.32 CuSO_4_; pH 5.5. The nutrient solution was replaced with fresh solution daily. NO_3_^−^ and NH_4_^+^ were supplied in the nutrient medium as Ca(NO_3_)_2_ and (NH_4_)_2_SO_4_. To exclude the potential effects of calcium (Ca^2+^) on the treatments, we supplemented the solutions in the same experimental system with Ca^2+^ (as CaCl_2_) to the levels experienced by plants under the highest NO_3_^−^ concentrations. The nitrification inhibitor dicyandiamide (7.0 μM) was added to each pot to prevent NH_4_^+^ oxidation.

### Measurement of root system architecture

The rice root system comprises seminal and adventitious roots, each bearing LRs^48^. We used previously-described LR developmental stage categories^3^; stages I–XII were grouped here into the “unemerged primordia” category. The primordia of LR were classified into unemerged and emerged primordia. An emerged LR primordium >0.5 mm long (visible to the unaided eye) was classified as a LR, and the primordium was considered to have been activated^9^.

To visualise the development of LRs, we stained the seminal roots with methylene blue^49^. These roots were fixed in formalin/acetic acid/alcohol (FAA) solution (10:5:85 v/v/v) at 4 °C for at least 24 h. After fixation, LR primordia were rinsed for 10 min in water and then stained with a 0.01% w/v methylene blue solution. After the roots had been stained, counting the numbers of LR primordia and LRs was straightforward. The scaleplate in the stereomicroscope we used (Olympus Optical Co. Ltd, Tokyo, Japan) simplified determinations of the lengths of emerged primordia and LRs. The lengths of seminal and adventitious roots were measured using a ruler, and LR density was calculated by dividing the LR number by root length. Total root length and LR length were measured using the WinRhizo scanner-based image analysis system (Regent Instruments, Montreal, QC, Canada).

### Lateral root responses to localised NO_3_^−^ supply

Rice seeds were germinated in trays over 2 d and then transferred into plant culture dishes (23 × 23 cm) containing 0.6% Phytagel™ media. Culture dishes were divided into three 5-cm segments; the upper and lower segments were supplemented with 0.2 mM NH_4_^+^, and the middle segment was supplemented with 0.2 mM NO_3_^−^. We photographed the representative morphologies of LRs in seminal roots after a 3 wk growth period. Each of the LR densities was calculated by dividing the LR number by the length of the seminal root segment.

The full chemical composition in the procedures using Phytagel medium was similar to that used in the hydroponic medium. To exclude the potential effects of calcium (Ca^2+^) on the treatments, the solutions in the three segments were supplemented with Ca^2+^ (as CaCl_2_) up to concentrations matching those in the NO_3_^−^ treatments.

### Measurement of ^15^N concentration

^15^N concentration was assayed in plants grown hydroponically^27^. After grinding in liquid N_2_, one aliquot of powder was dried to a constant weight at 70 °C. Approximately 6 mg powder from each sample were analysed using an Isotope Ratio Mass Spectrometer system (Thermo Fisher Scientific). To analyse the change in LR growth in response to the nutrient solution containing varying NO_3_^−^ concentrations, the same experiment without NO_3_^−^ labelling was performed at the same time. After harvest, LR number in the seminal root was analysed through recording of the LR number before and after the transfer of rice plants to nutrient solutions containing various NO_3_^−^ concentrations.

### Determination of IAA

We measured the concentrations of IAA in the first leaf and in the root^9^. Fresh weights of the samples were measured, after which specimens were immediately frozen in liquid N_2_. We performed sample measurement of free IAA by high-performance liquid chromatography. A standard IAA sample was obtained from Sigma-Aldrich (St. Louis, MO, USA).

The *pDR5::GFP* construct was transformed into WT plants and *osnar2*.*1* mutants using *Agrobacterium tumefaciens* (strain EHA105) to determine the patterns of IAA distribution in rice plants. To construct the *pDR5::GFP* vector, we amplified a 720-bp cDNA fragment of green fluorescent protein (GFP) from the cloning vector pSAT6A-EGFP-N1 using the following primer set: FP, GGATCCATGGTGAGCAA GGGCGAGGAGCT; RP, GAGCTCTCACTTGTACAGCTCGTCCATG. This fragment was inserted into the vector of *pDR5::GUS* at the BamHI and SacI sites. The *pDR5::GUS* construct was kindly provided by the Ping Wu laboratory at Zhejiang University, Hangzhou, China. We analysed the fluorescence of GFP in the cells using 543-nm helium-neon and 488-nm argon lasers using a confocal laser scanning microscope (LSM410; Carl Zeiss, Oberkochen, Germany).

### [^3^H]IAA-transport assay

Shoot-to-root auxin transport in intact plants was assayed^9^. WT plants and osnar2.1 mutants were pre-cultured for 1 wk in 0.2 mM NO_3_^−^ and NH_4_^+^ solutions. We applied 20 μL [^3^H] IAA solution to the cut surfaces after excision of rice shoots 4 cm above the root-shoot junction; plants were then kept in darkness for 18 h. The [^3^H] IAA solution applied contained 0.5 μM [^3^H] (20 Ci mmol^−1^) in 2% dimethylsulphoxide (DMSO), 25 mM 2-(N-morpholino) ethanesulphonic acid (MES) buffer (pH 5.2) and 0.25% agar. The root-shoot junction was dissected out and weighed before incubation in the scintillation solution for >18 h. [^3^H] IAA radioactivity was detected using a multipurpose scintillation counter (LS6500, Beckman-Coulter, Fullerton, CA, USA).

### Quantitative reverse transcription polymerase chain reaction (qRT-PCR) analysis

Total RNA was isolated from the roots of rice seedlings. RNA extraction, reverse transcription, and the qRT-PCR procedures followed a previously-reported procedure^50^. The primer sets targeting the PIN genes are listed in Supplementary Table S1.

### Data analysis

Data from experiments were pooled for calculation of means and standard errors (SE) and analysed by one-way ANOVA followed by the LSD test at *P* ≤ 0.05 to determine the statistical significance of the differences between individual treatments. All statistical evaluations were conducted using the SPSS (version 11.0) statistical software (SPSS Inc., Chicago, IL).

## Additional Information

**How to cite this article**: Huang, S. *et al.* Knockdown of the partner protein OsNAR2.1 for high-affinity nitrate transport represses lateral root formation in a nitrate-dependent manner. *Sci. Rep.* **5**, 18192; doi: 10.1038/srep18192 (2015).

## Supplementary Material

Supplementary Information
