# Supplementary material for: Knockdown of the partner protein OsNAR2.1 for high-affinity nitrate transport represses lateral root formation in a nitrate-dependent manner
Source: Sci Rep. 2015 Dec 8;5:18192. doi: 10.1038/srep18192 (PMC4672285; doi:10.1038/srep18192)
Supplement: Supplementary Information [file srep18192-s1.pdf]

Supplementary information

Knockdown of the partner protein OsNAR2.1 for high-affinity nitrate transport represses lateral root formation in a nitrate-dependent manner

Shuangjie Huang*, Si Chen*, Zhihao Liang, Chenming Zhang, Ming Yan, Jingguang Chen, Guohua Xu, Xiaorong Fan[+] & Yali Zhang[+]

State Key Laboratory of Crop Genetics and Germplasm Enhancement, Key Laboratory of Plant Nutrition and Fertilization in Low-Middle Reaches of the Yangtze River, Ministry of Agriculture, Nanjing Agricultural University, Nanjing 210095, P.R. China

*These authors contributed to this work equally.

Correspondence and requests for materials should be addressed to X.R.F. (xiaorongfan@njau.edu.cn) and Y.L.Z. (ylzhang@njau.edu.cn)

SupplementaryTable S1 The primers for qRT-PCR of OsPIN family genes

Gene	Primer sequence
OsPIN1a	5'-TCATCTGGTCGCTCGTCTGC-3'
	5'-CGAACGTCGCCACCTTGTTC-3'
OsPIN1b	5'-TGCACCCTAGCATTCTCAGCA-3'
	5'-CCCTCCTCCCAAATTCTACTT-3'
OsPIN1c	5'-CCGTCAGGTTCCTCGTGGGT-3'
	5'-TCACGGCTGTGCTCAGAATG- 3'
OsPIN1d	5'-GATTCCGACGTCGTCTCGCTCG-3'
	5'-GTCGGGTTCCGCGACGACTGCA-3'
OsPIN2	5'-CAACACCTACTCCAGCCTC-3'
	5'-TGGACCAGTCAAGAACCTC-3'
OsPIN5a	5'-GGGGCTGGTGCTAAAGTTCG-3'
	5'-TGAGGTAGGGCTGCCTGTATG-3'
OsPIN5b	5'-GGGCAGCAGGAGAGGGTGATAG-3'
	5'-GAATCGGCAGAGAGATCAATGT-3'
OsPIN5c	5'-CTTCACCGCCGACCAGTGCGAC-3'
	5'-GTGATGCACCACGAGAACCCGC-3'
OsPIN8	5'-GTTCCACTATATGTAGCTATGATAC-3'
	5'-CAGTCAAACTTCTCTGCACAGC-3'
OsPIN9	5'-GATACAAGATAGCGTCGTTCTC-3'
	5'-ATGATGTCTGCGTGGACCT-3'
OsPIN10a	5'-GTTGGATTGAGATAGGCTGAGGAG-3'
	5'-ATGGCGACGAAGCGGTTGAT-3'
OsPIN10b	5'-TCCGATGCAGGGTTAGGC-3'
	5'-AGGATGGTAGCGTGGAGGTT-3'

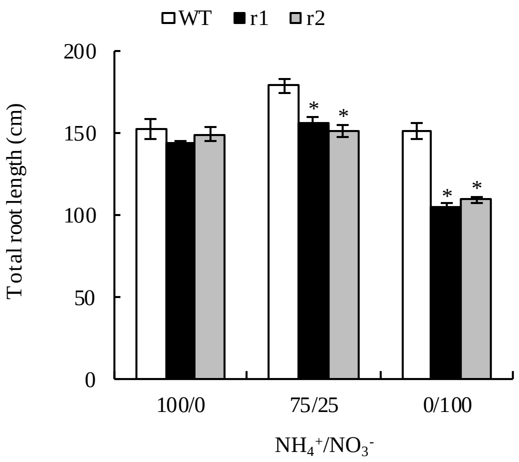

Supplementary Figure S1. Root morphology of the wild-type (WT) and *osnar2.1* knockdown lines (r1 and r2) under three NH_4^+/NO_3^- ratios at a total N concentration of 0.2 mM. Total root length was measured after the seedlings were grown for 1 wk in hydroponic media containing three ratios of NH_4^+/NO_3^-. Values are means ± SE (n = 6). *, $P < 0.05$ (ANOVA) comparing WT plants and two mutant lines in the same treatment.

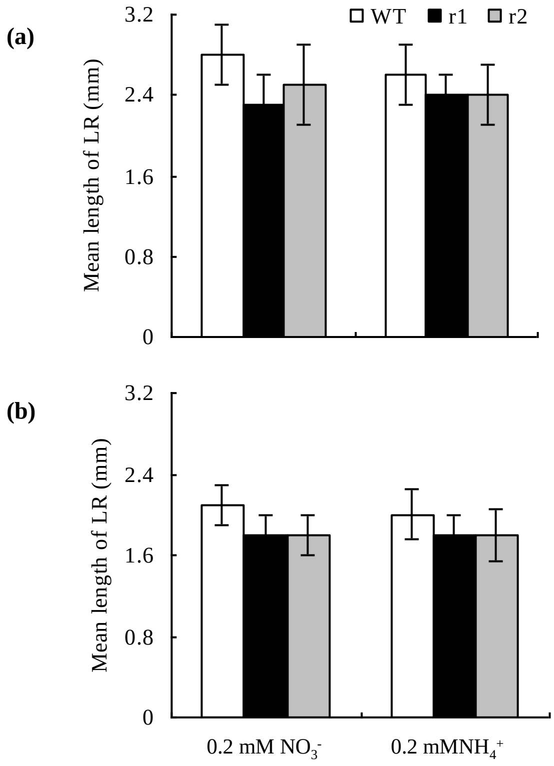

Supplementary Figure S2. Mean length of lateral root (LR) in seminal (a) and adventitious (b) roots in wild-type (WT) and *osnar2.1* knockdown lines (r1 and r2). Rice seedlings were grown for 1 wk in hydroponic media containing 0.2 mM NO_3^- or NH_4^+. Values are means ± SE (n=6).

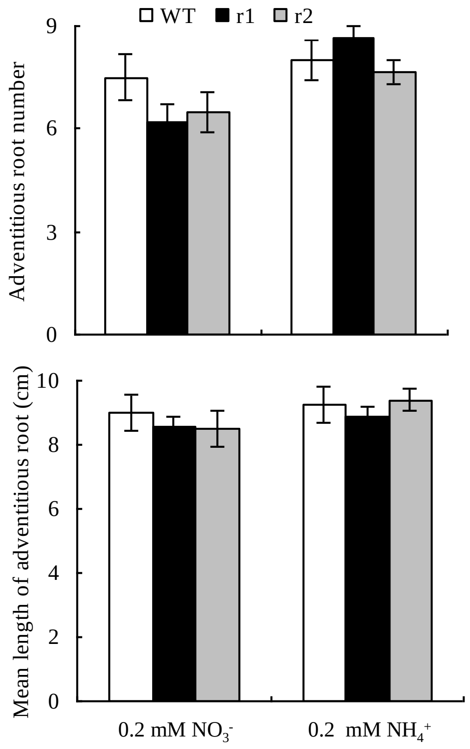

Supplementary Figure S3. Adventitious root number and mean adventitious root length in the wild-type (WT) and *osnar2.1* knockdown lines (r1 and r2). Rice seedlings were grown for 1 wk in hydroponic media containing 0.2 mM NO_3^- or NH_4^+. Values are means ± SE (n=6).
